# Development of a Serious Game for Training Multiple Types of Memory in Older Adults with Mild Cognitive Impairment

**DOI:** 10.1002/alz70861_108360

**Published:** 2025-12-23

**Authors:** Lucas Neves Santos, Lelio Moura Lourenço

**Affiliations:** ^1^ Universidade Federal de Juiz de Fora, Juiz de Fora, Minas Gerais Brazil; ^2^ Federal University of Juiz de Fora, Juiz de Fora Brazil

## Abstract

**Background:**

Mild cognitive impairment (MCI) is characterized by cognitive changes that are greater than expected for age but do not significantly interfere with daily activities. It is considered a risk condition for the development of dementia, highlighting the importance of early cognitive stimulation strategies. Memory training has proven effective in maintaining and potentially improving cognitive performance, especially when targeting different types of memory. Serious games emerge as innovative alternatives by combining motivational elements, structured repetition, and real‐time feedback. This study describes the development of *Memórias Vivas*, a digital serious game exclusively focused on training multiple types of memory in older adults with MCI, using the digital health intervention development framework as a structural guide.

**Method:**

Development follows five stages: (1) Theoretical and scientific foundation based on neuropsychological evidence related to memory and MCI, defining the types of memory to be stimulated (episodic, semantic, working, operational, and implicit); (2) Co‐design with healthcare professionals, caregivers, and end users to validate content, accessibility, and cultural relevance; (3) Design and development of minigames tailored to each memory type, featuring an accessible interface, multimodal stimuli, and intuitive mechanics; (4) Evaluation planning using standardized tests and qualitative data collection regarding user engagement and usability; (5) Dissemination strategy proposal for multiple platforms (Android and desktop), offering free access and guidance for home and institutional use.

**Result:**

Stage 1 has been completed with the development of a robust conceptual model aligned with scientific evidence on MCI and memory stimulation. Stage 2 is currently underway, with co‐design sessions involving professionals and users, whose input is being integrated to refine the game’s content, language, and aesthetic elements.

**Conclusion:**

Using structured frameworks supports the systematic and evidence‐based development of digital solutions centered on user needs. *Memórias Vivas* presents itself as a promising tool for training different types of memory in older adults with MCI, with potential to slow the progression of cognitive decline.

